# Design and development of a mobile application for drug information and other health data for users and patients of pharmacies and outpatient pharmaceutical services

**DOI:** 10.1016/j.rcsop.2025.100661

**Published:** 2025-09-21

**Authors:** Raquel Agudelo, Johan Granados, Mauricio Ceballos, Jaime Andrés Pereañez

**Affiliations:** a Grupo de Investigación de Promoción y Prevención Farmacéutica, Facultad de Ciencias Farmacéuticas y Alimentarias, Universidad de Antioquia, Medellín, Colombia; b Grupo de Investigación de Tecnología en Regencia de Farmacia, Facultad de Ciencias Farmacéuticas y Alimentarias, Universidad de Antioquia, Medellín, Colombia

**Keywords:** Pharmaceutical care, Information and communication technologies, Digital health, Mobile application, Outpatient pharmaceutical services, Usability

## Abstract

**Background:**

The advancement of Information and Communication Technologies (ICT) has transformed multiple sectors, including healthcare, by enhancing access to information, optimizing service management, and reshaping interactions between healthcare professionals and users.

**Objective:**

(1) To enhance the accessibility and quality of PC services, (2) to advance digital health implementation in Colombia by integrating pharmaceutical practice with innovative technological solutions that promote patient empowerment and patient- centered care models, (3) to demonstrate a comprehensive workflow for developing e- health applications using a User-Centered Design (UCD) approach.

**Methods:**

The study employed a User-Centered Design (UCD) approach, structured in four phases: (1) identification of user needs through an exploratory review and a descriptive study; (2) gathering and prioritization of functional requirements using the MoSCoW method; (3) design and development of a prototype based on six thematic databases; and (4) usability testing through task-based user evaluations and satisfaction rating scales.

**Results:**

A total of 72 functional requirements were identified and categorized into two main groups: those related to information provision and those linked to specific application features. Twenty functionalities were prioritized as essential, including drug information searches, healthy lifestyle guidance, medication intake reminders, and pharmacy geolocation. The final prototype comprised eight functional modules.Usability testing showed that 100 % of users completed most tasks, with task times under one minute and high satisfaction scores across all evaluated components.

**Conclusions:**

The application demonstrated high levels of acceptance and usability, establishing itself as a potentially effective tool to improve access to pharmaceutical care services, empower patients, and promote safe medication use in outpatient settings. Additionally, the methodology presented in this study may serve as a reference for the development of future digital health tools.

## 1 Introduction

The advancement of Information and Communication Technologies (ICTs) has revolutionized multiple disciplines, including healthcare, by enhancing information accessibility, optimizing service management, and transforming interactions between healthcare professionals and patients.^1^^,^^2^ In this digital health context, ICTs represent a strategic tool for strengthening patient-centered care, promoting self-management, and improving therapeutic outcomes.^3^^,^^4^ Notably, the field of pharmaceutical care (PC) has undergone significant evolution, expanding the pharmacist's role beyond simple medication dispensing and product inventory control^5^ to become a key player in comprehensive patient care.^6^, ^7^, ^8^

However, in developing countries, such as Colombia, significant challenges persist in accessing PC services, including fundamental components, such as education on the appropriate use of medication, especially in ambulatory settings.^9^, ^10^, ^11^ Although this country has achieved important advances in coverage and general access to health services,^12^ in Colombia, the healthcare system operates under a universal coverage model with both public and private providers. Pharmaceutical services are primarily delivered through retail pharmacies and outpatient services, with limited integration of clinical pharmacy services in ambulatory settings. Despite these structural arrangements, available information about accessibility and quality of PC services in outpatient contexts remains limited. The inadequate provision of these services not only reduces the impact of PC in preventing medication-related errors but also compromises patient safety and their active participation in the recovery process.^13^, ^14^, ^15^ Consequently, there is an urgent need to explore new strategies that contribute to overcoming geographical, economic, and structural barriers that impede equitable access to PC services.

In this context, ICTs emerge as a promising solution to bridge the gap between patients and PC services. The development of mobile applications integrating medication information, health education strategies, and functionalities related to appropriate medication use could enhance the pharmacist's role in outpatient settings by facilitating informed decision-making by patients and promoting therapeutic adherence. Nevertheless, despite the rapid advancement of health technologies, there is a scarcity of research specifically focused on designing digital solutions for the pharmaceutical environment in Colombia, which limits the full utilization of these tools.^16^

Given the identified need, the present study aimed to design and develop a mobile application that provides drug information and other health data to users and patients of pharmacies and outpatient pharmaceutical services. This initiative was designed with three objectives: (1) to enhance the accessibility and quality of PC services, (2) to advance digital health implementation in Colombia by integrating pharmaceutical practice with innovative technological solutions that promote patient empowerment and patient-centered care models, and (3) to demonstrate a comprehensive workflow for developing e-health applications using a User-Centered Design (UCD) approach.

## 2 Methods

### 2.1 Study design

This study developed a mobile application designed to provide medication information and health-related resources for patients of outpatient pharmaceutical services and pharmacies. The development process employed a user-centered design (UCD) approach to ensure end-user acceptability and usability. The research protocol comprised four phases described below.

#### 2.1.1 Phase I. Identification of user needs for appropriate medication use

This phase focused on identifying and characterizing needs related to the provision of PC services. An exploratory review of eHealth solutions oriented toward supporting these types of services was conducted to identify content, functionalities, and basic structures present in technological tools used in this field.^16^ Additionally, a descriptive cross-sectional study was conducted with patients from outpatient pharmaceutical services and pharmacies to understand their needs, characteristics, and preferences regarding the provision of PC services.^17^ This study was conducted in February and March 2024, involving 30 patients.

#### 2.1.2 Phase 2. Requirements gathering and design guidelines for the mobile application

During this phase, the application's functional requirements were defined. Based on the findings from the previous phase, an initial requirements list was developed, which was prioritized using the must have, should have, could have, and won't have (MoSCoW) methodology.^18^^,^^19^ This methodology classifies functionalities into four levels: must have, should have, could have, and won't have, according to their relevance, complexity, and feasibility within the project context.

Prioritization was based on two main criteria:

• Complexity, evaluated by a software engineer according to required technical effort, considering available tools and workload. A Likert scale from 1 (low complexity) to 5 (high complexity) was used.
• Feasibility, assessed by the research team, considering available resources (human, financial, and technological), development time, and environmental constraints. A Likert scale, ranging from 1 (low feasibility) to 5 (high feasibility), was also employed.

To each requirement was assigned a total score (sum of complexity and feasibility), and implementation priority was established according to the following scale^18^^,^^19^:

• 10 points: Must have (Priority 1).
• 8 to 9 points: Should have (Priority 2).
• 6 to 7 points: Could have (Priority 3).
• 0 to 5 points: Won't have (Priority 4).

This process enabled informed decisions about application development, focusing efforts on the most relevant and feasible functionalities within the project's conditions.

#### 2.1.3 Phase 3. Design and development of a graphical interface and an interactive prototype

In this phase, databases containing the application's information were structured, and the Scrum methodology was applied. It is an adaptive, lightweight framework for incremental software development by small, self-organizing teams.^20^ It uses time-boxed sprints, prioritized backlogs, and daily focused meetings led by a Scrum master, enabling rapid feedback, visibility, risk mitigation, and continuous stakeholder involvement.^20^^,^^21^

Based on requirements prioritized in the previous phase, six databases were constructed in Microsoft Excel files, corresponding to the following categories: drug monographs, commercial names, pathologies, healthy lifestyle habits, contraindicated drug interactions, and medication storage recommendations.

Information was collected from official and publicly accessible sources, including several aimed at the general population. These sources included: the regulatory agency from Colombia INVIMA's (Instituto Nacional de Vigilancia de Medicamentos y Alimentos) consultation portal for the Unique Medication Code (UMC) and renewal procedures, “Medicamentos a un clic,” the Colombian Ministry of Health Healthy Lifestyles site, the “Healthy Habits” section of the U.S. National Institutes of Health (NIH), and FDA (Food and Drug Administration)-approved medication technical data sheets.

Additionally, two Python scripts incorporating web scraping functions were designed and executed, allowing automation of part of the data collection process from medication URLs previously organized in an Excel file.

All databases were thoroughly reviewed, curated, and translated into clear, user-friendly language. This task was conducted by two members of the research team to ensure accuracy and consistency.

#### 2.1.4 Phase 4. Evaluation of initial prototype usability

A descriptive cross-sectional study was conducted with 10 users of outpatient pharmaceutical services and pharmacies, aged 18 to 70 years, who were literate. Non-probabilistic sampling was applied,^22^ and sample size was defined according to Nielsen's recommendations for usability testing.^23^

A specific instrument was designed to record participants' interactions with the application. This instrument collected data on sociodemographic characteristics, mobile device used, completion of assigned tasks, and questions about user preferences and satisfaction levels. To minimize bias, a combination of neutral open-ended and closed-ended questions was employed.

The test was conducted individually in an in-person setting, with prior informed consent obtained from all participants. Confidentiality and anonymity were strictly maintained throughout the study. The “Think Aloud” technique was employed,^24^ enabling participants to verbalize their thoughts during application use.

During the session, 11 tasks were assigned to evaluate tool performance. Subsequently, users rated different aspects of the application using a 1-to-5 Likert scale and provided additional comments. To minimize observer bias, it was emphasized that the evaluation focused on the application rather than the participants.

The analysis considered the success rate, defined as the percentage of tasks successfully completed, as well as efficacy measures, including required time to complete tasks, the number of necessary actions performed, and the frequency of errors committed during task execution.

## 3 Results

### 3.1 Phase I. Identification of user needs for appropriate medication use

#### 3.1.1 Exploratory review

Seven articles describing six applications focused on the provision of PC services were identified. Five of these applications were directed toward patients and were developed in different countries: Colombia,^25^ Germany,^26^ China,^27^ the United States,^28^ and Spain.^29^ Additionally, an eHealth platform in Greece was identified that integrates a mobile application for patients and a web tool aimed at pharmacists.^30^ For more details, see Agudelo et al. (2025).^16^

A total of 46 functions were identified from these six eHealth tools, which are oriented toward the delivery of outpatient PC services. Of these, ten corresponded with informational components, while the remaining 36 were associated with specific application functionalities. Among the most notable features were medication reminders, evaluation of therapeutic adherence, delivery of personalized information according to medical prescriptions (with or without direct interaction with a pharmacist), as well as information on appropriate medication use, potential side effects, and drug interactions (Supplementary material 1).

### 3.2 Descriptive study

The interviewed users demonstrated limited understanding of the functions of pharmaceutical services and pharmacies. While they reported using certain digital tools, none were associated with the provision of outpatient PC services. For more details, see Agudelo et al. (2025).^17^

Likewise, this study allowed the identification of a preliminary list of 38 needs related to the provision of PC services. These needs were classified into two main categories: informational needs and functional requirements of the mobile application. Among the most relevant were those related to access to information about appropriate medication use, disease education, contraindications, and information about healthy lifestyle habits (Supplementary material 2).

### 3.3 Phase 2. Requirements gathering and design guidelines for the mobile application

A total of 72 functional requirements were compiled, organized into two main categories: those related to information generation (Table 1) and those linked to specific application functionalities (Table 2, and Supplementary material 2).

**Table 1:** Functional requirements related to the mobile application information.

Requirement	Complexity (Likert Scale 1–5)	Feasibility (Likert Scale 1–5)	Total Sum	Priority	MoSCoW Classification
Information about the appropriate medication use (Administration with or without food)	5	5	10	1	Must have
Side effects	5	5	10	1	Must have
Drug interactions	5	5	10	1	Must have
Precautions and contraindications (Lactation)	5	5	10	1	Must have
Medication storage and conservation conditions	5	5	10	1	Must have
Information about pathologies	5	5	10	1	Must have
Healthy lifestyle (Healthy habits and diet information)	5	5	10	1	Must have
Approved indication	5	5	10	1	Must have
Routes of administration	5	5	10	1	Must have
Medication trademarks	5	5	10	1	Must have
Medication composition	4	4	8	2	Should have
Nearby pharmacies	3	5	8	2	Should have
Safety and effectiveness parameters information	5	0	5	4	Won't have
Antidotes in case of intoxication	5	0	5	4	Won't have
Price references	5	0	5	4	Won't have
Medication availability	5	0	5	4	Won't have
Posology (Dose and frequency of administration)	0	5	5	4	Won't have
Clinical history	2	0	2	4	Won't have
List of prescribed medications, vaccination, physicians, medication history and prescriptions	2	0	2	4	Won't have
Medication delivery location	2	0	2	4	Won't have
Delivery waiting time	2	0	2	4	Won't have
Treatment duration	2	0	2	4	Won't have
Out-of-stock medications	2	0	2	4	Won't have
Available pharmaceutical forms	2	0	2	4	Won't have
Mechanisms of action	2	0	2	4	Won't have
Therapeutic alternatives	0	0	0	4	Won't have
Age-appropriate use	0	0	0	4	Won't have
Allergy recommendations	0	0	0	4	Won't have
Onset time of pharmacological effect	0	0	0	4	Won't have
Non-pharmacological treatments	0	0	0	4	Won't have

**Table 2:** Functional requirements related to specific mobile application functions.

Requirement	Complexity (Likert Scale 1–5)	Feasibility (Likert Scale 1–5)	Total Sum	Priority	MoSCoW Classification
Provision of general and open medication information	5	5	10	1	Must have
Text search command by generic and trademark name	5	5	10	1	Must have
Medication registration: manual or by scanning	5	5	10	1	Must have
Addition of frequently consulted medications	5	5	10	1	Must have
Search for nearby hospitals and pharmacies through GPS	5	5	10	1	Must have
Medication reminders and medication use notes	5	5	10	1	Must have
BMI calculator	5	5	10	1	Must have
Allergy registration	5	5	10	1	Must have
Contraindication alerts	5	5	10	1	Must have
General information consultation for prescribed medications (Use, ADRs, Interactions)	5	5	10	1	Must have
Periodic sending of educational scientific dissemination messages	0	5	5	4	Won't have
Periodic sending of medication follow-up questionnaires to specific patients to collect adherence, effectiveness and ADR data	0	5	5	4	Won't have
Medication schedule	2.5	2.5	5	4	Won't have
ADR identification	5	0	5	4	Won't have
ADR registration	2.5	2.5	5	4	Won't have
ADR and interaction verification functions	5	0	5	4	Won't have
Motivational videos for therapy adherence	5	0	5	4	Won't have
Vibration and sound alerts	0	4	4	4	Won't have
Voice command (Voice assistant)	2	2	4	4	Won't have
Automatic message sending with prescribed medication information	2	0	2	4	Won't have
Treatment completion and home medication stock reminders	2	0	2	4	Won't have
Appointment request for medication renewal	2	0	2	4	Won't have
Direct communication with office, physician, pharmacy and health administrator	2	0	2	4	Won't have
Registration of diseases or surgical interventions performed	2	0	2	4	Won't have
Registration of temperature, blood pressure and weight	2	0	2	4	Won't have
Questionnaires related to oncological therapy and pain	2	0	2	4	Won't have
Medication delivery scheduling	2	0	2	4	Won't have
Automatic clinical information collection	2	0	2	4	Won't have
Unification of medication authorization and renewal	2	0	2	4	Won't have
Alert to physician to renew prescription	2	0	2	4	Won't have
Font size adjustment	4	0	4	4	Won't have
Fagerstrom scoring test for nicotine addiction	4	0	4	4	Won't have
Provision of personalized information according to medical prescriptions - with or without pharmacist interaction	0	0	0	4	Won't have
Therapeutic compliance evaluation	0	0	0	4	Won't have
Barcode capture	0	0	0	4	Won't have
Therapeutic compliance registration and information transfer to pharmacist	0	0	0	4	Won't have
Multiple user profiles	0	0	0	4	Won't have
Online communication with pharmacist for medication consultation services	4	0	4	4	Won't have
Communication with pharmaceutical personnel through text or voice	4	0	4	4	Won't have
Chat Bot and medication schedule calendar	0	0	0	4	Won't have
Behavioral skills measurement	0	0	0	4	Won't have
Tooltips that accompany the process	0	0	0	4	Won't have

Of these, 30 requirements were classified as informational and 42 as functional. In terms prioritization, 20 requirements were assigned as Priority 1, evenly distributed between the two categories (10 informational and 10 functional). Additionally, two requirements were assigned Priority 2, both related to information generation. No requirements were classified under Priority 3. Finally, 50 requirements were assigned Priority 4, of which 32 corresponded to application functionalities and 18 to information generation.

### 3.4 Phase 3. Design and development of a graphical interface and an interactive prototype

Six databases were structured to consolidate the information provided by the mobile application. The characteristics of the databases are detailed below.

1. Drug monographs: It included information on 563 active principles and 2082 ambulatory medications, detailing the international nonproprietary name (INN), concentration, pharmaceutical form, dosage, route of administration, indications, use, precautions, contraindications, side effects, special populations, and reference sources.
2. Trademark names: This database contained 3167 trademarked medication names currently available on the market, each linked to its corresponding active ingredient as registered in the monographs database.
3. Pathologies: This database comprises 71 diseases grouped into 14 categories, with information on description, risk factors, therapeutic objectives, general management recommendations, and references. Among the included conditions are digestive, autoimmune, gynecological, endocrine, and musculoskeletal diseases.
4. Healthy lifestyles: It offered recommendations on physical activity, nutrition, weight control, smoking prevention, oral and auditory health, stress management, and responsible alcohol consumption, each with its respective information source.
5. Contraindicated drug interactions: It recorded 54 clinically relevant interactions, supported by excellent and good quality evidence.
6. Medication storage recommendations: This database included guidance on home conservation, management of medications requiring a cold chain, appropriate disposal conditions, and warnings about use after deterioration or expiration.

**Fig. 1:**
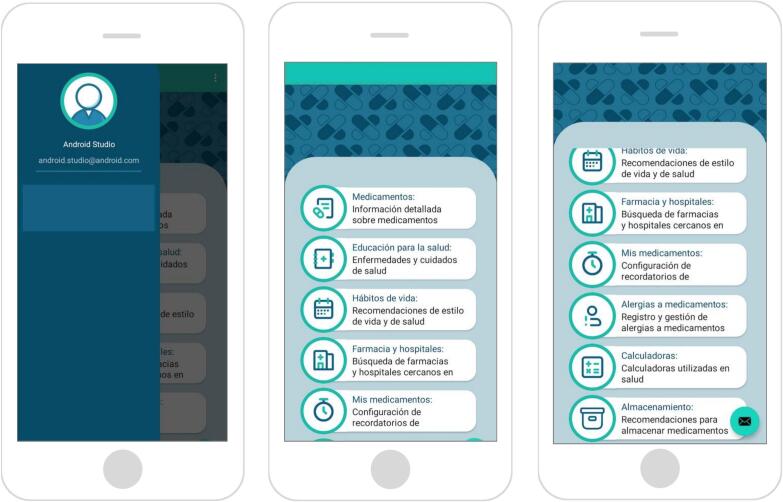
Main menu and search options of the initial prototype.

Based on the functional requirements prioritized at levels 1, 2, and 3, a prototype was developed comprising eight modules. Medications, Health education, Healthy lifestyles, Nearby pharmacies and hospitals, My medications, Medication allergies, Calculators, and Storage. (Fig. 1). The specific characteristics of each module are described in detail below.

An initial prototype was designed with an attractive and user-friendly graphical interface that appropriately combined colors, contrasts, font sizes, and icons, optimized for the end-user experience (Fig. 1). The prototype included a medication search option by trademark or generic name, which allowed access to detailed information such as concentration, pharmaceutical form, dosage form, route of administration, indications, use, precautions, contraindications, side effects, special populations, references, and therapeutic class (Supplementary material Fig. 1S).

A “Disease consultation” module was also incorporated, allowing for the visualization of information related to a disease description, risk factors, treatment objectives, and general management guidelines (Supplementary material Fig. 2S).

Similarly, the “Healthy lifestyles” module provided access to evidence-based content on various health-promoting topics, including physical activity, balanced nutrition, body weight control, tobacco consumption prevention, oral health, auditory health, stress management, and risks associated with alcohol consumption. Each topic was supported by corresponding references (Supplementary material Fig. 3S).

Additionally, the prototype included a function that enabled users to locate nearby pharmacies and hospitals based on their geographic location. This functionality required both an internet connection and GPS activation. The information is provided through integration with Google Maps, allowing users to independently visualize the locations of healthcare facilities on the map. Furthermore, a list displayed at the bottom of the screen presented complementary details, including distance, address, operating hours, and directions to each facility (Supplementary material Fig. 4S).

The “My medications” module allowed users to add frequently used or chronic medications, providing easy access to their information without the need for repeated searches. It also included a configurable reminder system, enabling users to activate or deactivate according to preferences. Additionally, the system alerted the user about contraindicated interactions if they registered two medications that are not recommended to be administered simultaneously (Supplementary material Fig. 5S).

The “Allergy registration” module allowed users to input information via free-text entry, organized into two categories: “Medication or Food” and “Allergy manifestation” (Supplementary material Fig. 6S). In addition, the calculators module supported the computation of key health indicators, including body mass index (BMI), waist-to-hip ratio, and basal energy expenditure. These calculations are performed by entering the required data and selecting the corresponding calculation function (Supplementary material Fig. 7S).

Finally, the “Storage” module provided general recommendations on the appropriate storage and disposal of medications (Supplementary material Fig. 8S).

### 3.5 Phase 4. Evaluation of initial prototype usability

The usability test was conducted with a total of 10 participants, comprising four men and six women. The median age was 38 years, with an interquartile range (IQR) of 29.3 years.

Each participant was informed about the study and subsequently downloaded the application onto their mobile devices. Under the guidance of a facilitator, participants completed a series of 11 tasks, during which their preferences and any observed difficulties were documented.

Overall, task success rates were high, with nine out of eleven tasks achieving a 100 % completion rate. The remaining two tasks, Task 8 (Access usage information for a registered medication in “My Medications”) and Task 9 (Remove a medication from the list in “My Medications”) achieved success rates of 80 % and 90 %, respectively. Median times per task were less than one minute. Tasks T1 and T8 showed the highest number of errors, whereas T5, T6, and T10 were completed without any errors (Table 3).

**Table 3:** Task completion, time, and errors.

Task	% Completed	Time (sec) Median (IQR)	# of errors
T1 - Search for medication information by INN	100	55.0 (33.8)	10
T2 - Search for medication information by trademark name	100	41.0 (20.0)	4
T3 - Search for pathology information	100	37.5 (33.8)	4
T4 - Search for healthy lifestyle information	100	10.0 (7.68)	1
T5 - Search for appropriate medication storage information	100	10.5 (10.0)	0
T6 - Search for nearby pharmacies and hospitals	100	10.0 (10.8)	0
T7 - In “My medications” register medication and configure alarm for dose reminder	100	31.5 (12.8)	5
T8 - In “My medications” access usage information for registered medication	80	15.0 (12.0)	10
T9 - In “My medications” remove medication from medication list	90	10.0 (13.0)	5
T10 - Register allergy	100	27.1 (8.80)	0
T11 - Calculate BMI	100	24.0 (24.4)	5

Additionally, during the test, the facilitator digitally recorded observations regarding user difficulties, suggestions, and preferences when performing assigned tasks. These data facilitated the identification of critical usability issues.

Key findings revealed functional deficiencies, including: an inability to edit medication administration time in “My medications” module (users were forced to delete and recreate alerts rather than modify existing entries), users failed to access the information of registered medications. Inaccurate BMI calculations due to unit inconsistencies (the system expected height input in meters but accepted centimeters without conversion, resulting in erroneous outputs). Opportunities for interface improvement were also identified, including the need for more intuitive icons, improved text presentation (misspelled words), and options to organize information by group.

User satisfaction was evaluated through two questions. The first, regarding intention to use, revealed that 100 % of participants would use the application to consult medication use, highlighting its usefulness and the clarity of the information offered.

Additionally, a Likert scale (1 = Very poor, 5 = Very good) was applied to assess the general functioning of the application. The aspects evaluated included:

• Ease of use.
• Accessibility.
• Content and information quality.
• Design and graphical interface.
• Search functions (by INN/Trademark, pathology, and healthy lifestyles).
• Map search.
• Medication configuration.
• Allergy registration.
• BMI calculator.
• Storage recommendations.

**Fig. 2:**
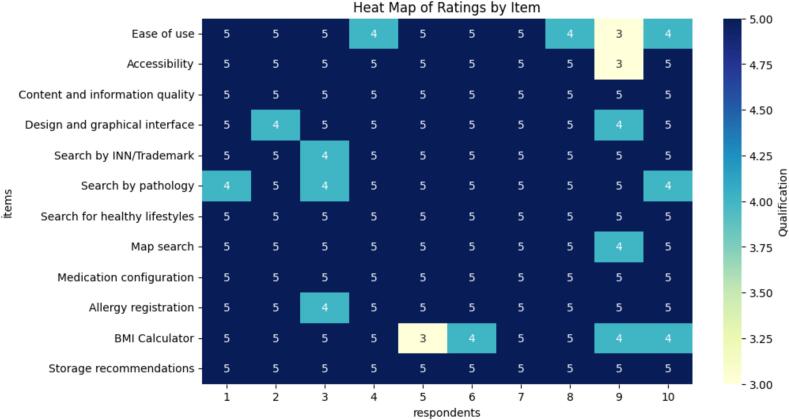
General application rating.

The results indicated a positive perception by users in all evaluated components (Fig. 2).

It is important to note that this app did not collect any user data; all information remained securely stored on the user's mobile device.

## 4 Discussion

Limited access to PC services remains a significant obstacle to the appropriate use of medication, impacting therapeutic effectiveness, patient safety, and incurring additional costs for healthcare systems.^31^, ^32^, ^33^, ^34^, ^35^ To address this critical gap, this study developed an app to facilitate patient access to reliable information about medications and key aspects related to their use. This tool was created to empower users in making informed decisions, improve therapeutic adherence, and ultimately enhance the delivery of pharmaceutical care services.

The development was based on the UCD approach, as recommended by the World Health Organization.^36^ This methodology allowed the incorporation of users' needs, expectations, and perceived barriers from the early stages. Various studies have demonstrated that UCD improves usability, functionality, and acceptance of digital tools,^37^, ^38^, ^39^, ^40^ which is essential in the case of digital solutions that seek to complement and expand the scope of PC services.

In contrast to previously reviewed applications, which focus exclusively on appropriate medication use,^26^, ^27^, ^28^, ^29^, ^30^ this proposal offers a comprehensive approach to healthcare. In addition to medication information (including indications, contraindications, precautions, storage, and use in special populations), the app also integrates educational content about healthy habits, tools for calculating anthropometric indicators such as BMI and waist-to-hip ratio, and a drug interactions alert system.

A relevant functionality of the application is georeferencing, which enables users to locate nearby pharmacies and healthcare facilities. This module improves patient orientation within the health system and facilitates timely access to PC services, especially in contexts where geographical dispersion represents a barrier. Consistent with the findings of Madrigal et al.,^25^ the implementation of this feature was highly valued by users, who emphasized its practical utility and positive impact on accessibility.

Although the proposal could not be integrated with institutional clinical information systems due to technical and methodological limitations, it is recognized that greater interoperability would allow linking these developments with pharmacotherapeutic follow-up processes, pharmacovigilance, and personalized pharmaceutical education. Several authors agree that this integration is fundamental for strengthening PC services from a person-centered model.^27^^,^^29^^,^^30^ In the Colombian context, advancing toward this type of solution requires a more robust political, technical, and regulatory environment that supports the digital transformation of the pharmaceutical health *sec*tor.^36^

Finally, this development experience highlights the potential of mobile technologies to expand and complement the delivery of PC services, particularly in resource-constrained environments. In alignment with international digital health guidelines, the findings highlight the importance of designing solutions based on real user needs, adapted to the local context, and aligned with the broader goal of achieving universal health coverage.^36^ In this way, studies like this contribute to improve equitable access to quality pharmaceutical services through accessible, reliable, and person-centered technological tools.

## 5 Limitations

This study could have some methodological limitations that need to be kept in mind when thinking about user-centered design research. It's worth saying that usability studies and needs assessments are not really meant to prove statistical generalization, since the main goal is to create a working application, not to make broad predictions. Because of that, the number of participants used (30 for needs assessment and 10 for usability testing) follows what is normally accepted in UCD methodology and also what Nielsen suggests for usability testing.^23^^,^^24^ The purpose here is more about finding user needs and practical usability issues, not reaching strict statistical representativeness.

The main limitations of this study have to do with scope and time. The development only worked on Android devices because of resource limits, which left iOS users out of the evaluation. Also, the research was carried out in a very specific geographic and healthcare context (Antioquia, Colombia). The usability test was done in just one single session, without long-term follow-up, so it doesn't show if people would keep using it or how effective it could be in real-life pharmaceutical care settings. On top of that, technical and regulatory challenges stopped the evaluation of integration with existing healthcare information systems, which is something quite important if the solution is going to be implemented and scaled in the future.

## 6 Conclusion

A mobile application designed to provide information about medications and other relevant health data was developed, targeting users of retail pharmaceutical establishments and ambulatory pharmaceutical services. The development was based on the UCD methodology, which allowed for the involvement of end users from the initial stages through to project completion. Throughout the process, not only were user needs identified about these services, but also, through prototype evaluation, design problems were identified and corrected to improve the user experience.

## CRediT authorship contribution statement

**Raquel Agudelo:** Writing – review & editing, Writing – original draft, Visualization, Methodology, Investigation, Formal analysis, Data curation, Conceptualization. **Johan Granados:** Writing – review & editing, Writing – original draft, Visualization, Methodology, Investigation, Formal analysis, Conceptualization. **Mauricio Ceballos:** Writing – review & editing, Investigation, Funding acquisition. **Jaime Andrés Pereañez:** Writing – review & editing, Writing – original draft, Methodology, Investigation, Formal analysis.

## Ethical considerations

The study was approved by the Ethics Committee of the Faculty of Nursing of the Universidad de Antioquia, Colombia (Minutes No. CEI-FE 2022-20).

## Declaration of competing interest

The authors declare that they have no known competing financial interests or personal relationships that could have appeared to influence the work reported in this paper.

## Appendix A Supplementary data

Supplementary material 1 Figures 1S-8S. Image 1

Supplementary material 2 Image 2
