# Supplementary material for: Design and development of a mobile application for drug information and other health data for users and patients of pharmacies and outpatient pharmaceutical services
Source: Explor Res Clin Soc Pharm. 2025 Sep 21;20:100661. doi: 10.1016/j.rcsop.2025.100661 (PMC12510017; doi:10.1016/j.rcsop.2025.100661)
Supplement: Supplementary file 1 — Supplementary material 1 Figures 1S-8S. [file mmc1.zip › mmc1.pptx]

## Slide 1
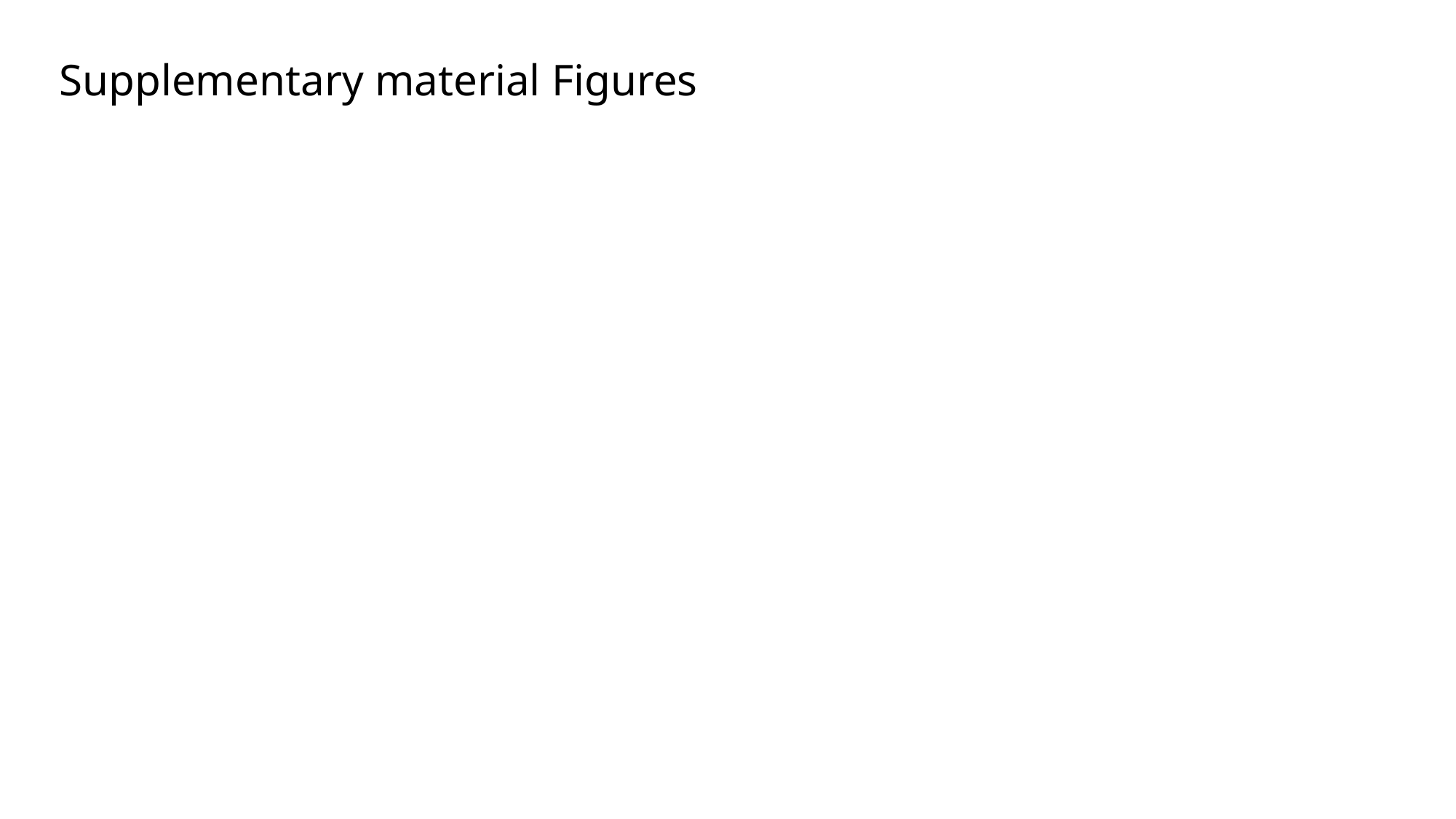

Supplementary material Figures

## Slide 2
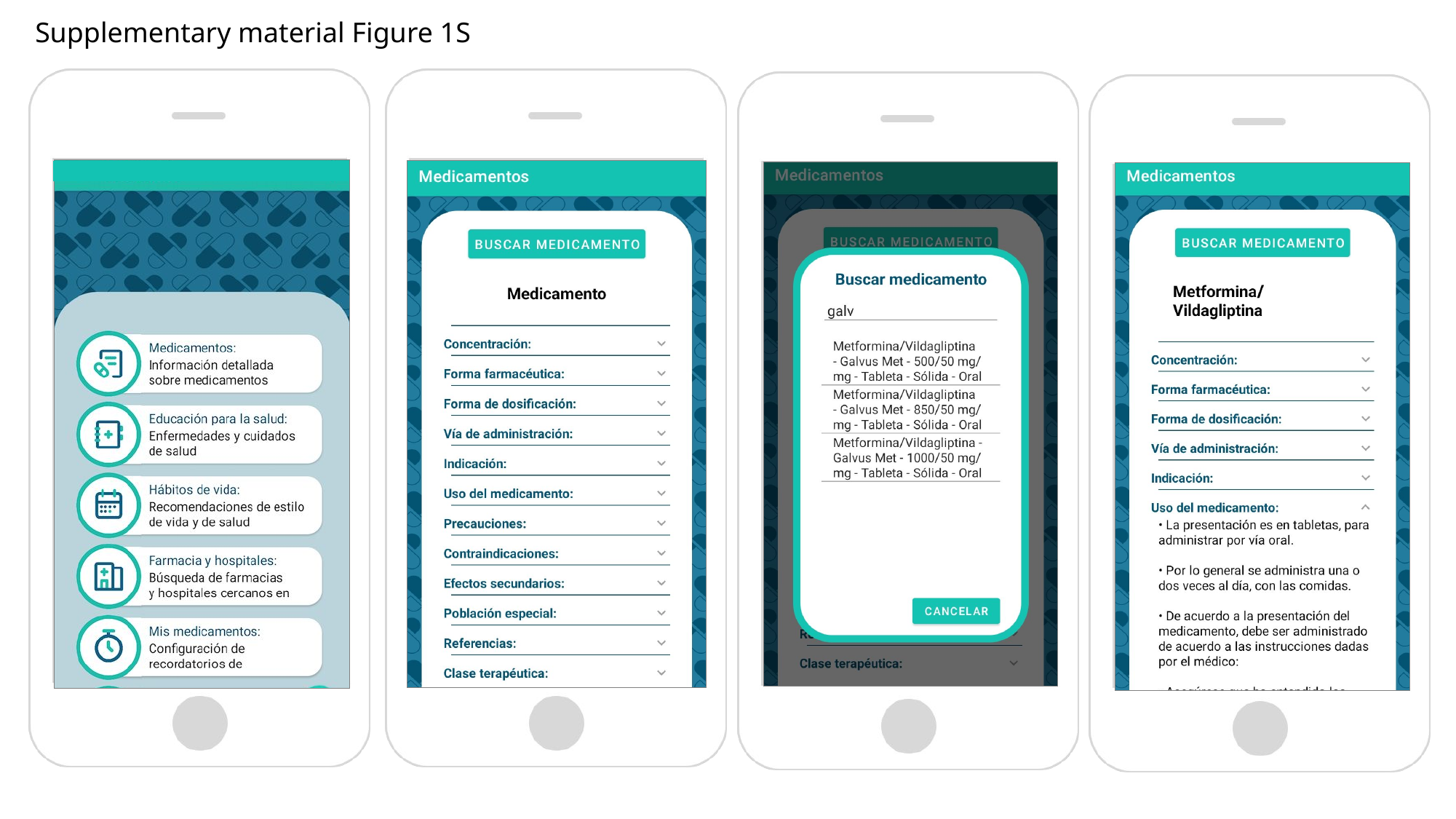

Supplementary material Figure 1S

## Slide 3
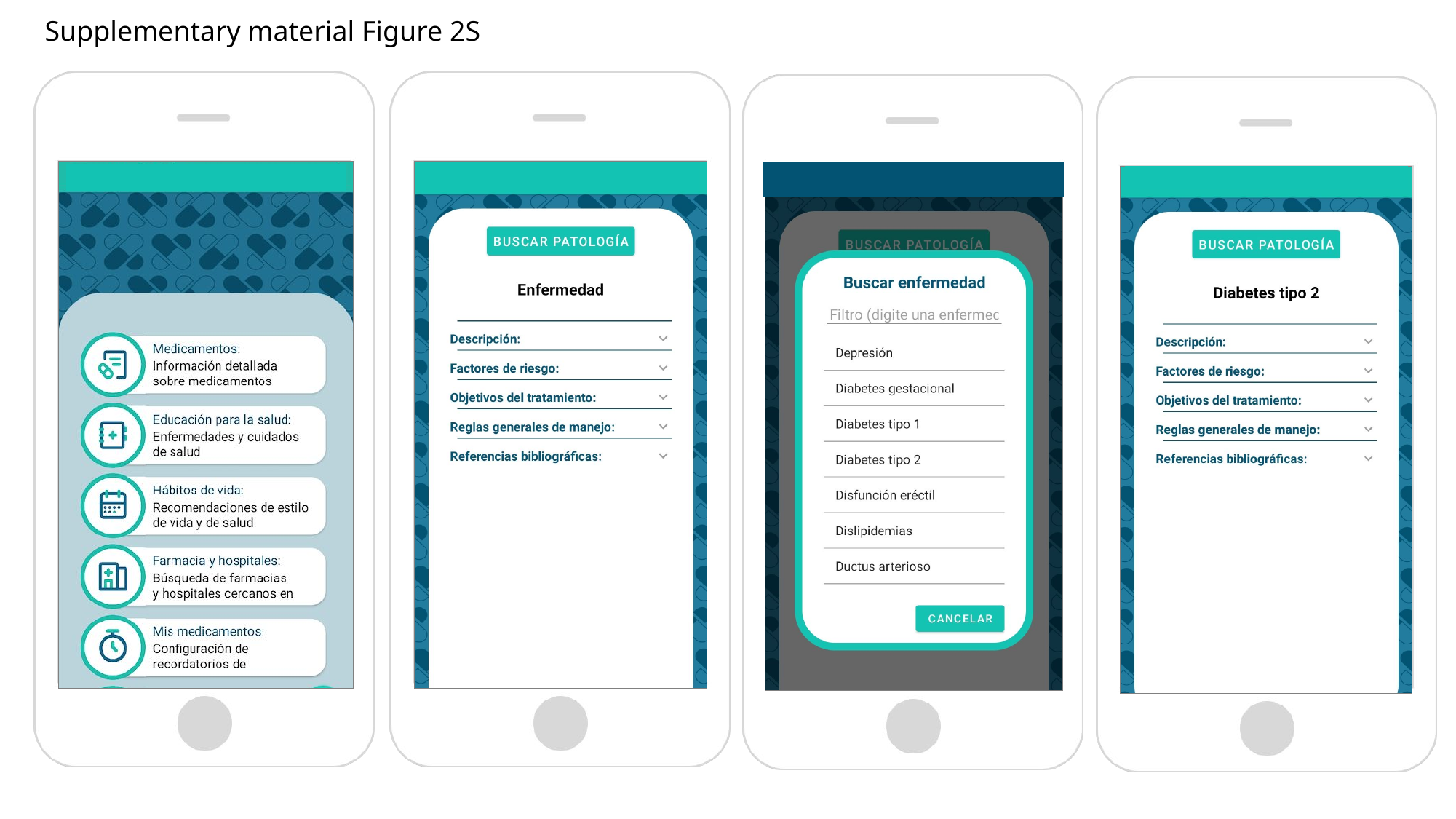

Supplementary material Figure 2S

## Slide 4
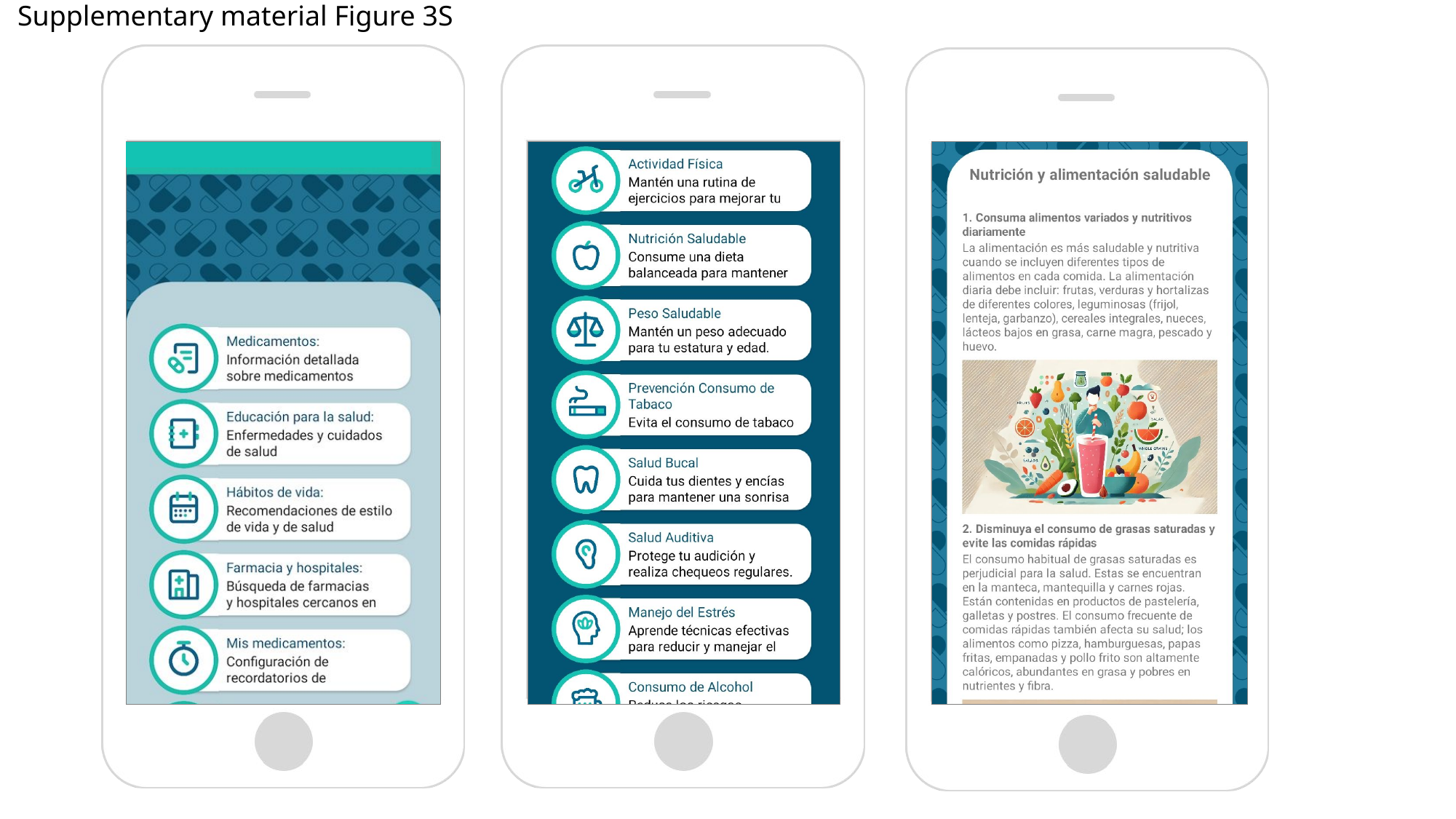

Supplementary material Figure 3S

## Slide 5
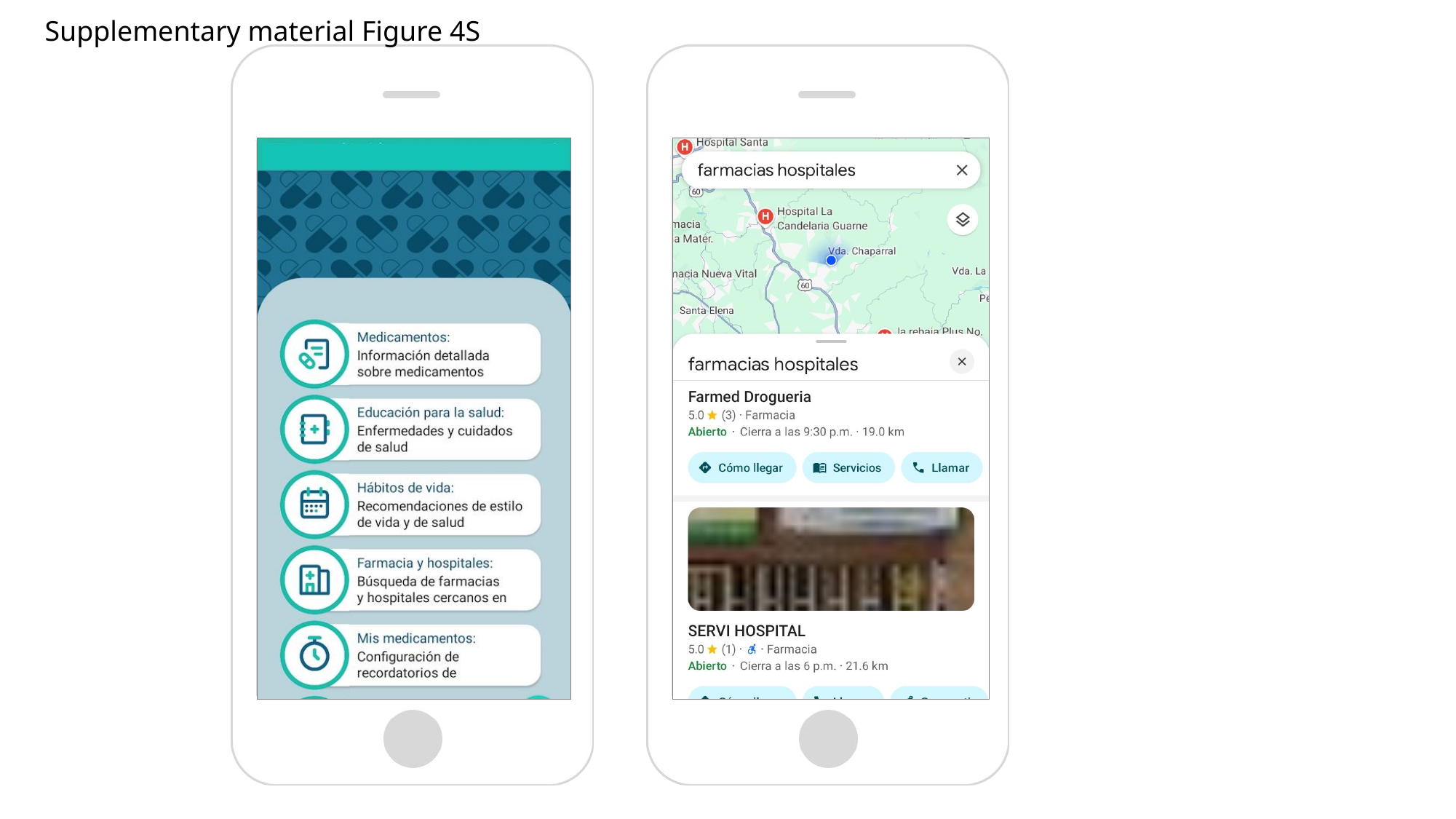

Supplementary material Figure 4S

## Slide 6
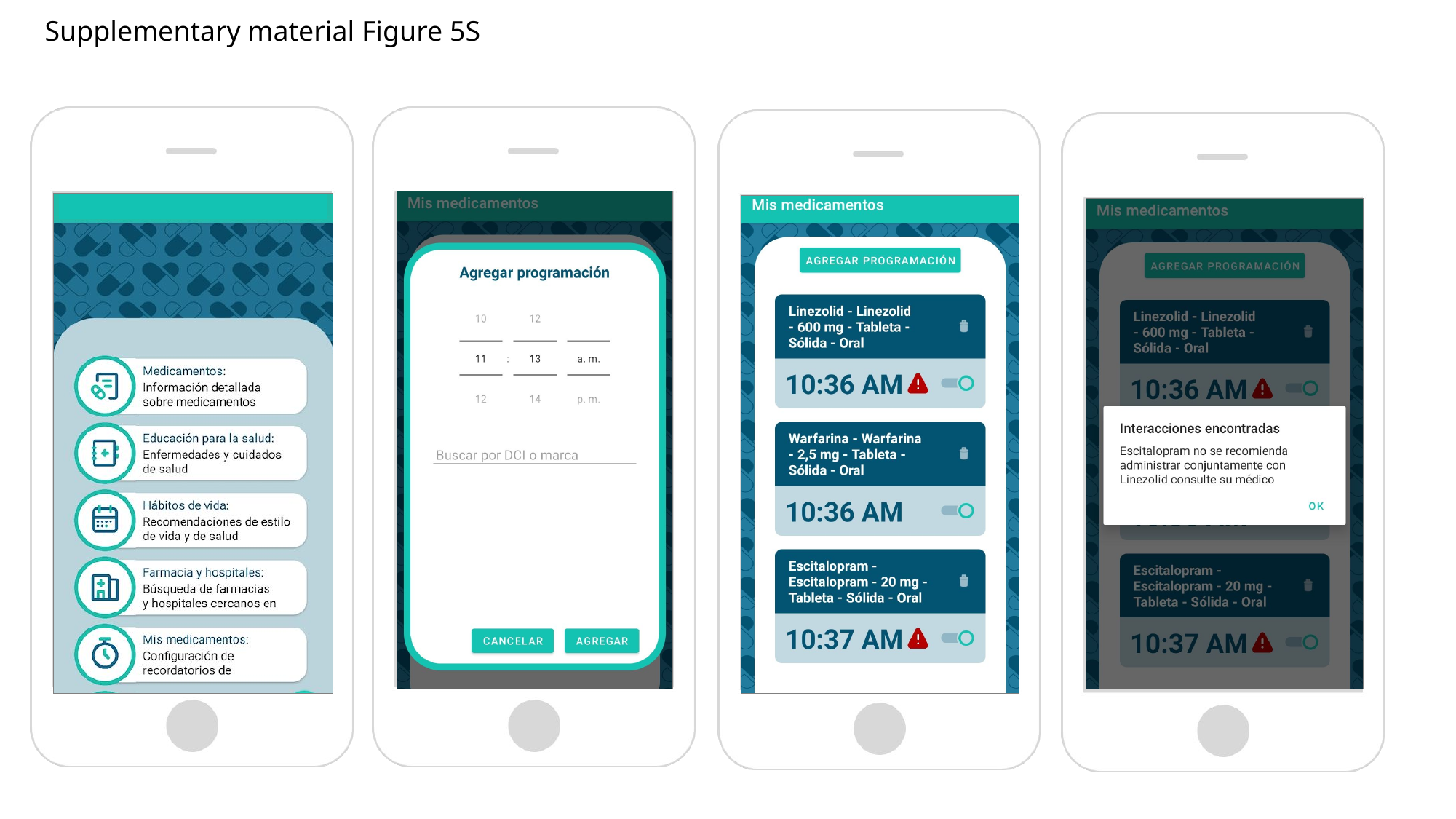

Supplementary material Figure 5S

## Slide 7
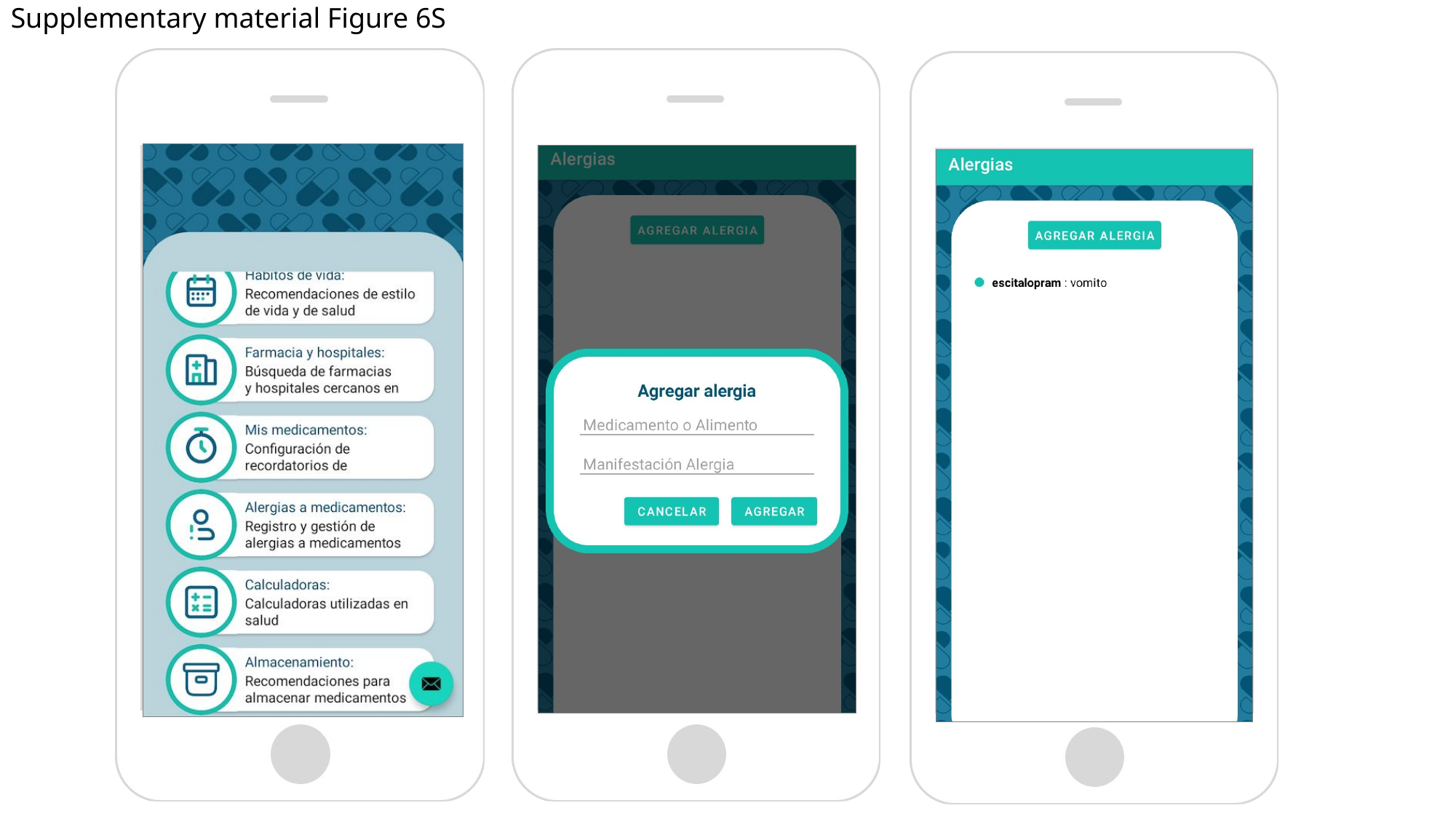

Supplementary material Figure 6S

## Slide 8
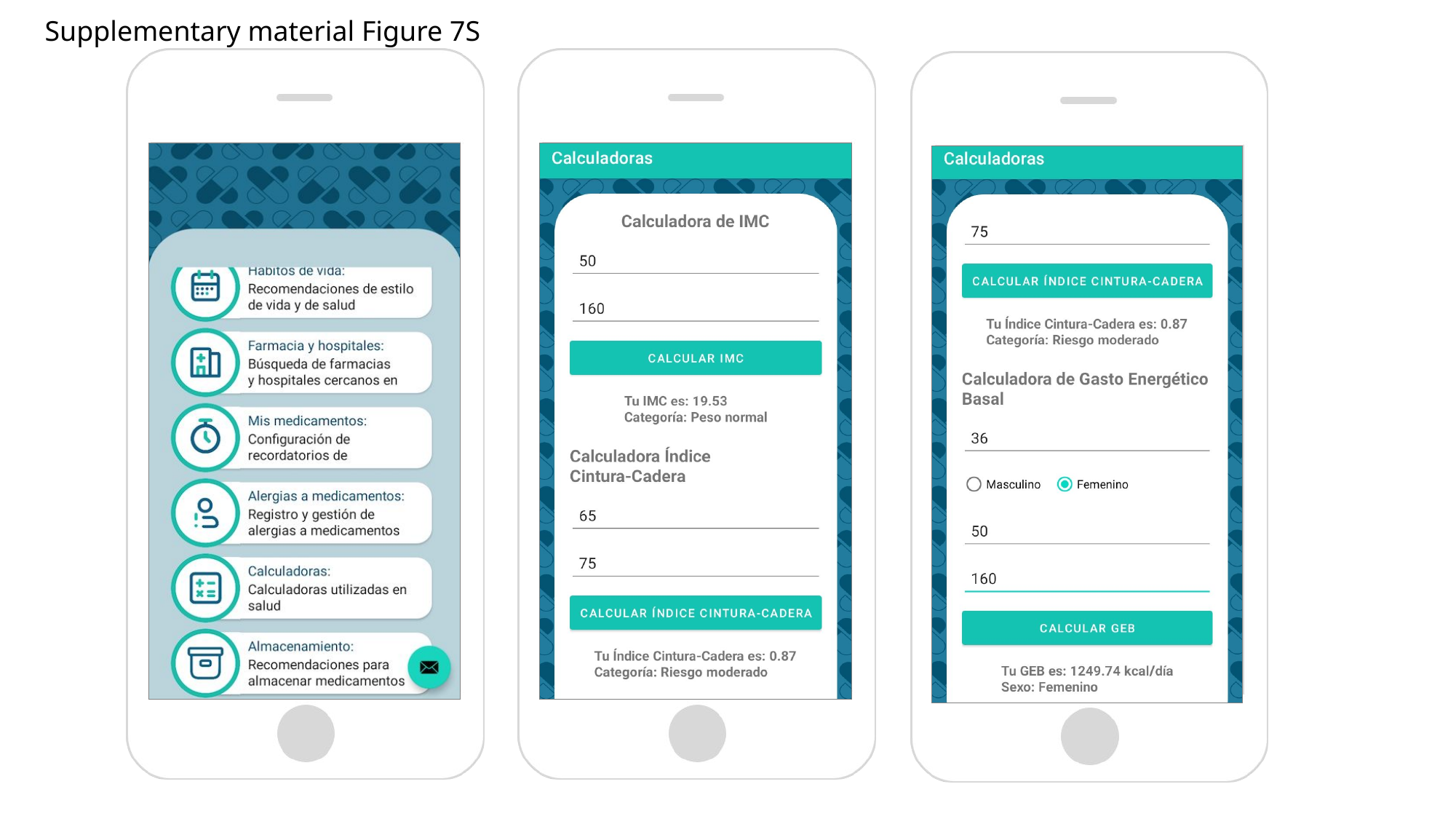

Supplementary material Figure 7S

## Slide 9
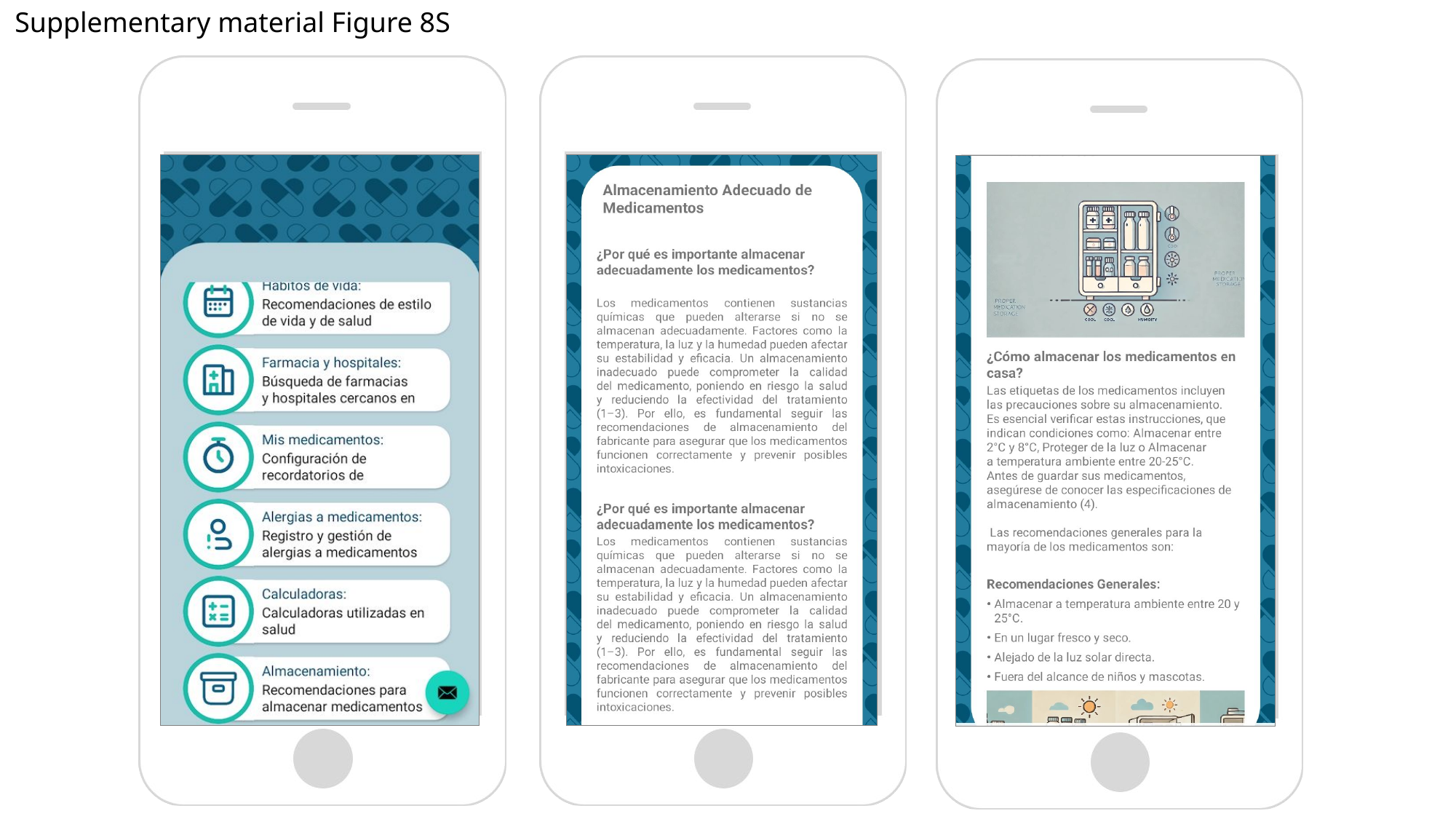

Supplementary material Figure 8S
