# Supplementary material for: Design and development of a mobile application for drug information and other health data for users and patients of pharmacies and outpatient pharmaceutical services
Source: Explor Res Clin Soc Pharm. 2025 Sep 21;20:100661. doi: 10.1016/j.rcsop.2025.100661 (PMC12510017; doi:10.1016/j.rcsop.2025.100661)
Supplement: Supplementary file 2 — Supplementary material 2 [file mmc2.docx]

**Supplementary Material 1. List of functionalities identified in mobile applications focused on the provision of professional pharmaceutical services**

|  | | **FarmaceuticApp** | **PharmActa** | **Sedlmayr B, et al, 2019** | **Xiang Medicine Guidance (XMG)** | **My Personal Health Guide** | **SERGAS-MOBIL** |  |
| --- | --- | --- | --- | --- | --- | --- | --- | --- |
| **Information-related** | | | | |  |  |  |  |
|  | 1. Usage information | ✓ | - | - | ✓ | ✓ | - |  |
|  | 1. Adverse effects | ✓ | ✓ | - | ✓ | - | - |  |
|  | 1. Drug interactions | ✓ | ✓ | - | ✓ | - | - |  |
|  | 1. Information about pathologies | - | - | - | - | ✓ | - |  |
|  | 1. Information about safety and effectiveness parameters | - | - | - | - | ✓ | - |  |
|  | 1. Precautions and contraindications | ✓ | - | - | ✓ | - | - |  |
|  | 1. Storage and conservation conditions for medications | ✓ | - | - | ✓ | - | - |  |
|  | 1. Healthy lifestyle | - | ✓ | - | ✓ | - | - |  |
|  | 1. List of prescribed medications, vaccination, physicians | - | ✓ | - | - | - | - |  |
|  | 1. Antidotes in case of intoxication | - | ✓ | - | - | - | - |  |
|  |  |  |  |  |  |  |  |  |
| **Functionality-related** | | | | | | | |  |
|  | 1. Therapeutic compliance registration and information transfer to pharmacist | - | ✓ | - | - | - | - |  |
|  | 1. Therapeutic compliance assessment | - | ✓ | - | ✓ | - | ✓ |  |
|  | 1. Provision of personalized information according to medical prescription - With or without pharmacist interaction | ✓ | - | - | ✓ | - | - |  |
|  | 1. Provision of general and open drug information | - | ✓ | ✓ | - | - | - |  |
|  | 1. ADR identification | - | ✓ | - | ✓ | - | - |  |
|  | 1. Font size adjustment | ✓ | - | - | ✓ | - | - |  |
|  | 1. Voice commands (Voice assistant) | ✓ | - | - | ✓ | - | - |  |
|  | 1. Medication registration /// Manual, scanning medication registration /// Treatment registration | ✓ | - | ✓ | - | - | - |  |
|  | 1. ADR registration | - | ✓ | - | - | - | - |  |
|  | 1. Vibration and sound alerts | ✓ | - | - | - | - | - |  |
|  | 1. Barcode capture | ✓ | - | - | - | - | - |  |
|  | 1. Text search command by generic and brand name | ✓ | - | - | - | - | - |  |
|  | 1. Addition of frequently consulted medications | ✓ | - | - | - | - | - |  |
|  | 1. Search for nearby hospitals and pharmacies through GPS | ✓ | - | - | - | - | - |  |
|  | 1. General information consultation of prescribed medications (Use, ADR, Interactions) | - | ✓ | - | - | - | - |  |
|  | 1. Medication reminders and medication usage notes | - | ✓ | ✓ | ✓ | - | ✓ |  |
|  | 1. ADR and interactions verification functions | - | ✓ | - | - | - | - |  |
|  | 1. BMI calculator | - | ✓ | - | - | - | - |  |
|  | 1. Fagerstrom score test for nicotine addiction | - | ✓ | - | - | - | - |  |
|  | 1. Multiple user profiles | - | ✓ | - | - | - | - |  |
|  | 1. Automatic message sending with information about prescribed medications | - | - | - | ✓ | - | - |  |
|  | 1. Treatment completion reminder and home medication stock | - | - | - | ✓ | - | - |  |
|  | 1. Appointment request for medication renewal | - | - | - | ✓ | - | - |  |
|  | 1. Online communication with pharmacist for medication consultation services | - | - | - | ✓ | - | - |  |
|  | 1. Communication with pharmacy staff through text or voice |  |  |  | ✓ |  |  |  |
|  | 1. Periodic sending of educational messages for scientific dissemination | - | - | - | ✓ | - | - |  |
|  | 1. Periodic sending of medication follow-up questionnaires to specific patients, to collect adherence, effectiveness and ADR data | - | - | - | ✓ | - | - |  |
|  | 1. ChatBot and medication calendar | - | - | - | - | ✓ | - |  |
|  | 1. Direct communication with clinic, pharmacy and health administrator /// Communication with physician | - | - | - | - | ✓ | - |  |
|  | 1. Behavioral skills measurement | - | - | - | - | ✓ | - |  |
|  | 1. Motivational videos for therapy adherence | - | - | - | - | ✓ | - |  |
|  | 1. Summary of each medication | - | - | ✓ | - | - | - |  |
|  | 1. Tooltips that accompany the process | - | - | ✓ | - | - | - |  |
|  | 1. Registration of diseases or surgical interventions performed | - | - | ✓ | - | - | - |  |
|  | 1. Registration of temperature, blood pressure and weight | - | - | - | - | - | ✓ |  |
|  | 1. Questionnaires related to oncological therapy and pain | - | - | - | - | - | ✓ |  |
|  |  |  |  |  |  |  |  |  |

**Supplementary Material 2. List of perceived needs by users and patients of retail pharmaceutical establishments and outpatient pharmaceutical services**

|  | | **Droguería y Farmacia-Droguería**  **n** | **Servicio Farmacéutico**  **n** | **Total**  **n** |
| --- | --- | --- | --- | --- |
| **Information they would like to have available** | | | | |
|  | 1. Medication usage | 6 | 10 | 11 |
|  | 1. Disease education | 5 | 6 | 11 |
|  | 1. Contraindications/including lactation /// Precautions for use | 3 | 4 | 7 |
|  | 1. Healthy habits/Dietary information | 3 | 4 | 7 |
|  | 1. Posology (Dose and administration frequency) | 4 | 1 | 5 |
|  | 1. Approved indication | 2 | 2 | 4 |
|  | 1. Therapeutic alternatives | 2 | 2 | 4 |
|  | 1. Effectiveness indicators | 1 | 2 | 3 |
|  | 1. Medication availability | - | 3 | 3 |
|  | 1. Medication/formula history | 1 | 2 | 3 |
|  | 1. Adverse effects | 2 | - | 2 |
|  | 1. Administration routes | 1 | 1 | 2 |
|  | 1. Medication composition | 1 | 1 | 2 |
|  | 1. Treatment duration | - | 2 | 2 |
|  | 1. Allergy registration | 2 | - | 2 |
|  | 1. Drug interactions | - | 1 | 1 |
|  | 1. Clinical history | - | 1 | 1 |
|  | 1. Medication delivery location | - | 1 | 1 |
|  | 1. Available pharmaceutical forms | 1 | - | 1 |
|  | 1. Mechanisms of action | 1 | - | 1 |
|  | 1. Nearby sales points | 1 | - | 1 |
|  | 1. Delivery waiting time | - | 1 | 1 |
|  | 1. Age-appropriate usage | - | 1 | 1 |
|  | 1. Medication schedule | 1 | - | 1 |
|  | 1. Medication brands | 1 | - | 1 |
|  | 1. Allergy recommendations | - | 1 | 1 |
|  | 1. Pharmacological effect onset time | 1 | - | 1 |
|  | 1. Out-of-stock medications | - | 1 | 1 |
|  | 1. Price references | 1 | - | 1 |
|  | 1. Non-pharmacological treatments | 1 | - | 1 |
|  | |  |  |  |
| **Functionalities they would like to have available** | | | | |
|  | 1. Provision of personalized information according to medical prescription - With or without pharmacist interaction //// Personalized information | - | 1 | 1 |
|  | 1. Medication registration /// Manual, scanning medication registration /// Treatment registration | 1 | - | 1 |
|  | 1. Direct communication with clinic, pharmacy and health administrator /// Communication with physician | - | 1 | 1 |
|  | 1. Medication delivery scheduling | - | 1 | 1 |
|  | 1. Automatic collection of clinical information | - | 1 | 1 |
|  | 1. Unification of authorization and medication renewal | - | 1 | 1 |
|  | 1. Contraindication alerts | - | 1 | 1 |
|  | 1. Physician alert for prescription renewal | - | 1 | 1 |
|  |  |  |  |  |
